# A Novel Striated Muscle-Specific Myosin-Blocking Drug for the Study of Neuromuscular Physiology

**DOI:** 10.3389/fncel.2016.00276

**Published:** 2016-12-01

**Authors:** Dante J. Heredia, Douglas Schubert, Siddhardha Maligireddy, Grant W. Hennig, Thomas W. Gould

**Affiliations:** Department of Physiology and Cell Biology, University of Nevada School of MedicineReno, NV, USA

**Keywords:** neuromuscular, neurodegenerative, fatigue

## Abstract

The failure to transmit neural action potentials (APs) into muscle APs is referred to as neuromuscular transmission failure (NTF). Although synaptic dysfunction occurs in a variety of neuromuscular diseases and impaired neurotransmission contributes to muscle fatigue, direct evaluation of neurotransmission by measurement of successfully transduced muscle APs is difficult due to the subsequent movements produced by muscle. Moreover, the voltage-gated sodium channel inhibitor used to study neurotransmitter release at the adult neuromuscular junction is ineffective in embryonic tissue, making it nearly impossible to precisely measure any aspect of neurotransmission in embryonic lethal mouse mutants. In this study we utilized 3-(N-butylethanimidoyl)-4-hydroxy-2H-chromen-2-one (BHC), previously identified in a small-molecule screen of skeletal muscle myosin inhibitors, to suppress movements without affecting membrane currents. In contrast to previously characterized drugs from this screen such as N-benzyl-p-toluene sulphonamide (BTS), which inhibit skeletal muscle myosin ATPase activity but also block neurotransmission, BHC selectively blocked nerve-evoked muscle contraction without affecting neurotransmitter release. This feature allowed a detailed characterization of neurotransmission in both embryonic and adult mice. In the presence of BHC, neural APs produced by tonic stimulation of the phrenic nerve at rates up to 20 Hz were successfully transmitted into muscle APs. At higher rates of phrenic nerve stimulation, NTF was observed. NTF was intermittent and characterized by successful muscle APs following failed ones, with the percentage of successfully transmitted muscle APs diminishing over time. Nerve stimulation rates that failed to produce NTF in the presence of BHC similarly failed to produce a loss of peak muscle fiber shortening, which was examined using a novel optical method of muscle fatigue, or a loss of peak cytosolic calcium transient intensity, examined in whole populations of muscle cells expressing the genetically-encoded calcium indicator GCaMP3. Most importantly, BHC allowed for the first time a detailed analysis of synaptic transmission, calcium signaling and fatigue in embryonic mice, such as in *Vamp2* mutants reported here, that die before or at birth. Together, these studies illustrate the wide utility of BHC in allowing stable measurements of neuromuscular function.

## Introduction

Impaired synaptic transmission is observed at early stages of many neurodegenerative and auto-immune diseases of the central and peripheral nervous systems (Shankar and Walsh, 2009; Milnerwood and Raymond, 2010; Kayser and Dalmau, 2011). For example, at the neuromuscular junction (NMJ), the peripheral synapse between motor neurons (MNs) and skeletal muscle, disruption of synaptic transmission leads to diminished motor function in auto-immune diseases such as Lambert-Eaton Myasthenic Syndrome (LEMS; Kaja et al., 2007) and Myasthenia Gravis (MG; Serra et al., 2012) or inherited ataxias such as episodic ataxia 2 (EA2; Maselli et al., 2003). Similarly, deterioration of synaptic transmission occurs in advance of denervation and axon degeneration in neurodegenerative diseases such as spinal motor atrophy (SMA; Martinez et al., 2012) and amyotrophic lateral sclerosis (ALS; Shahidullah et al., 2013). Impaired neurotransmission also is a feature of muscular dystrophy (MD; van der Pijl et al., 2016) and inherited peripheral neuropathies such as Charcot-Marie Tooth disease Types 1 and 2 (CMT1, 2; Yin et al., 2004; Spaulding et al., 2016) Recently, we observed functional defects in the absence of denervation in an animal model of congenital hypomyelinating neuropathy (CHN; Scurry et al., 2016). Other forms of peripheral neuropathy such as type 2 diabetes also exhibit defective neuromuscular transmission (Allen et al., 2015). Finally, disruptions of synaptic transmission contribute to the reduction in muscle force caused by continued muscle activity, commonly referred to as muscle fatigue (Bazzy and Donnelly, 1993). Therefore, deciphering the mechanisms of impaired neuromuscular transmission is an integral component of therapeutic strategies aimed at restoring neuromuscular function in disease.

A failure to convert presynaptic neuronal action potentials (APs) to postsynaptic muscle APs is referred to as neural transmission failure (NTF). NTF may result from a failure of the neuronal action potential to propagate down the axon to presynaptic terminals, a reduction of the release of neurotransmitter by presynaptic terminals, an impairment of postsynaptic sensitivity to neurotransmitter, or a disruption of the propagation of the muscle action potential along the sarcolemma (Sieck and Prakash, 1995). The mechanisms of NTF have been explored in studies of high-frequency nerve stimulation (HFS), which result in synaptic depression caused by a reduction or rundown in the release of neurotransmitter (Eccles, 1943; Otsuka et al., 1962). For example, presynaptic features contributing to synaptic depression include a reduction in the number of synaptic vesicles released (quantal content) or in the amount of neurotransmitter per synaptic vesicle (quantal size; Heuser et al., 1979; Naves and Van der Kloot, 2001). The reduction of vesicle release underlying synaptic depression is caused by the depletion of vesicles in the ready releasable pool (RRP), a population of synaptic vesicles docked to the presynaptic membrane and released first upon nerve stimulation (von Gersdorff and Matthews, 1997).

At the vertebrate NMJ, the electrophysiological measurement of neurotransmitter (acetylcholine; ACh) release is performed by the recording of synaptic membrane potential or current in response to nerve stimulation. This technique is facilitated by the use of μ-conotoxin IIIb (μ-CTX), which inhibits the skeletal muscle-specific, voltage-gated Na_v_1.4 channel and therefore isolates the ACh receptor-mediated endplate current or potential (EPC, EPP) from the Na_v_1.4-mediated action potential (AP; Hong and Chang, 1989). In addition, this drug paralyzes the muscle, permitting the electrophysiological study of HFS on transmitter release by limiting muscle movement. Coupled with studies of muscle force decline in response to similar paradigms of high-frequency nerve vs. muscle stimulation, the relative contribution of NTF to fatigue has been assessed (Kuei et al., 1990).

Although μ-CTX is a useful tool to measure ACh release at the vertebrate NMJ, it cannot be used to directly determine the amount of ACh required to trigger muscle contraction (i.e., the level needed to avoid NTF), because the threshold for Na_v_1.4 channel activation is less than the amount of ACh released in response to nerve stimulation and measured in the presence of μ-CTX (Rich, 2006). This discrepancy, referred to as the safety factor, ensures that modest disruptions of ACh release fail to prevent neuromuscular signal transmission. Therefore, in order to assess what level of diminished ACh release is sufficient to fall below the safety factor and trigger NTF, the amount released must be measured and compared to the amount required to open Na_v_1.4 channels. This threshold has been measured by comparing muscle potential waveforms elicited by nerve stimulation in the presence of paralytic vs. sub-paralytic doses of the ACh receptor-binding toxin curare (Wood and Slater, 2001). Whereas sub-maximal, paralytic curare doses exclusively produce an EPP waveform, subparalytic doses produce a compound waveform of the EPP and the AP, and the point of AP inflection represents the Na_v_1.4 activation threshold. Using these approaches, the safety factor in rodent diaphragm has been estimated as 1.7–5, suggesting that NTF emerges when EPP amplitudes fall to as little as 58% of their original values (Wood and Slater, 2001).

Measuring NTF in this fashion is indirect, technically difficult and also produces movement, fracturing electrodes. Moreover, because Na_v_1.4 channels are blocked, the parameters of the unattenuated muscle AP itself cannot be assessed in response to nerve stimulation. Although muscle stimulation-evoked APs have been measured in teased fiber bundles that are physically manipulated to prevent movement (Delbono and Kotsias, 1987), or in response to single nerve-evoked APs (van Lunteren and Moyer, 1998), this approach has limited utility in the study of neuromuscular function. Because a wide variety of cell-specific gene overexpression or deletion models, as well as neurodegenerative and auto-immune disease models, are available in mice, the ability to inhibit contractility for the evaluation of APs in this animal model would be a powerful tool to elucidate the molecular mechanisms of NTF. With such a tool, NTF could be directly measured as failed muscle APs, whose height would be dramatically less than successful APs, based on the all-or-nothing nature of the muscle AP. Moreover, the study of factors affecting AP waveform parameters could be easily investigated. Muscle calcium dynamics could also be evaluated in response to nerve stimulation with such a drug, in contrast to studies using μ-CTX, because movement but not the release of calcium from intracellular stores would be blocked. Finally, the analysis of neurotransmission in embryonic mouse mutants could be assessed for the first time, since Na_v_1.5 channels, which are resistant to μ-CTX (Wilson et al., 2011), are expressed in fetal muscle, rather than μ-CTX-sensitive Na_v_1.4 channels (Lupa et al., 1993). Recently, a drug originally identified in a skeletal muscle-specific myosin ATPase-inhibitor screen, *N*-benzyl-*p*-toluene sulfonamide (BTS; Cheung et al., 2002), was used to measure muscle APs and ionic currents in adult mouse skeletal muscle cells Wang et al., 1999; Woods et al., 2004), as well as to measure nerve stimulation-induced muscle APs at the cane toad NMJ (Etherington et al., 2014).

In order to determine if BTS or other small molecules could inhibit skeletal muscle movement without affecting neurotransmission in mice tissue, we examined their effect in hemidiaphragm preparations. We show that although BTS is unable to inhibit mouse diaphragm contractility without also inhibiting phrenic nerve-induced release of ACh, a second drug identified but not characterized in the myosin ATPase screen, 3-(N-butylethanimidoyl)-4-hydroxy-2H-chromen-2-one (heretofore referred to as BHC), effectively blocks muscle movement without affecting nerve function. First, we characterize the effects of BHC on nerve-evoked APs. We then utilize BHC to compare the relative time to predicted AP failure (in the presence of μ-CTX) to the actual time of AP failure (NTF; in the presence of BHC) in response to HFS. Next, we compare the rates of HFS that cause NTF with those that cause muscle fatigue, using conventional and novel methods to measure muscle force. Using transgenic mice expressing GCaMP3, we then employ BHC to examine the effects of HFS on calcium signaling in muscle fibers. GCaMP3 is a fusion protein between the cytosolic calcium-sensing protein calmodulin and the fluorescent reporter GFP (Tian et al., 2009). In the absence of calcium, the fluorescence of GCaMP3 is inhibited, whereas in response to an increase of cytosolic calcium, a conformational shift occurs, and the protein fluoresces, producing a calcium transient. Finally, we demonstrate that BHC blocks muscle movement in response to nerve stimulation during embryonic development, allowing for the measurement of nerve stimulation-induced muscle APs and calcium signaling. Using BHC, we show that NTF occurs prematurely and fatigue is enhanced in *synaptobrevin 2*/*Vamp2* mutants, which die immediately after birth. Together, these studies introduce BHC as an experimental tool that permits the study of multiple new features of neuromuscular transmission during embryogenesis and in the context of disease.

## Materials and methods

### Ethical approval and use of mice

Animal husbandry and experiments were performed in accordance with the National Institutes of Health *Guide for the Care and Use of Laboratory Animals* and the Institutional Animal Use and Care Committee at the University of Nevada. *Vamp2* mutant mice were purchased from Jax. CAGGS-GCaMP3 transgenic mice were kindly provided by S. Pfaff (Salk Institute, San Diego, CA, USA). Each of these mice was backcrossed several times into the Balb/C background. For studies with adults, control Balb/C mice were sacrificed at 3–4 months of age. For studies of embryos, females were timed bred with males, and noon on the day of the plug was designated E0.5. After cervical dislocation of the dam, embryos were quickly dissected with their placentas intact and placed in oxygenated (95% O_2_ and 5% CO_2_) Krebs-Ringer's solution, tails were cut and processed for genotyping (e.g., Vamp2 mutants) by incubating in 100 μl of 10 N NaOH / 0.2 mM EDTA at 100 degrees for 15 min, followed by neutralization with 100 μl of 40 mM Tris, pH 5, and diluted 10-fold in water before being used as a template for PCR. During the PCR, diaphragms from every littermate were dissected and placed in changes of freshly oxygenated Krebs-Ringer's solution every 30 min.

### Drugs

BHC (Catalog number 5102862; Hit2Lead/Chembridge, San Diego, CA, USA) was dissolved in DMSO at 100 mM and frozen in aliquots at −20. Four μl of this stock was then added to 16 μl of DMSO, which was then bath applied to a Sylgard-coated 6-ml dish containing 8 mls of Krebs-Ringer's solution for 30 min without perfusion. This resulted in concentrations of 50 μM BHC and 0.05% DMSO. The bath was then perfused for 30 min before recording or imaging experiments commenced. BTS (TCI Chemicals, Portland, OR, USA) was dissolved in DMSO and used at the concentrations indicated. The Na_v_1.4 antagonist μ-conotoxin GIIIb (μ-CTX; Peptides International, Louisville, KY, USA) was dissolved in water and used at a final concentration of 2.3 μM. Finally, 3-(N-butylethanimidoyl)-4-hydroxy-2H-chromen-2-one (Catalog number 5102863; Hit2Lead/Chembridge) was used at 50 μM in similar fashion to BHC.

### Myosin heavy chain immunohistochemistry

The central region of the costal portion of the left diaphragm muscle was dissected from adult mice that had been anesthetized with isoflurane. The tissue was then rinsed in PBS and immediately fresh-frozen in a cryomold containing OCT (Tissue-Tek, Sakura, CA) in 2-methylbutane on dry ice. The tissue was cut transversely at 16 μm and immediately immersed in IgG blocking reagent (1/100 in PBS) for 30 min at room temperature (RT; 21°C) and then protein diluent (1/100 in PBS) for 30 min at RT, both from the mouse-on-mouse (MOM) kit (Vector Labs, Burlingame, CA, USA). Mouse primary antibodies (BA-F8 against myosin heavy chain (MHC) Type I; SC-71 against MHC Type IIA, and BF-F3 against Type IIB) were added at 1/100 concentration into protein diluent solution overnight at four degrees, rinsed then incubated with mouse isotype-specific fluorescent secondary antibodies as described (Bloemberg and Quadrilatero, 2012). We were unable to produce staining with the 6H1 antibody against Type IIX-expressing fibers, but utilized the slight background obtained with Type IIB staining to identify and count negative cells as Type IIX.

### Electrophysiology

Whole diaphragm muscles were dissected and pinned on a Sylgard-coated dish containing oxygenated Krebs-Ringer's solution at RT as described (Scurry et al., 2016). After 30 min of perfusion, the left phrenic nerve was drawn into a suction electrode and stimulated with supra-maximal square waves from an SD9 stimulator (2–5 V, 0.1 ms for adults; 4–10 V, 0.1 ms for embryos) or from an S48 stimulator coupled to a SIU5 stimulus isolation unit (both from Grass, Quincy, MA, USA). Sharp intracellular recording electrodes were made with tip resistances of ~30–60 MΩ and backfilled with 2 M potassium citrate and 10 mM potassium chloride. Correct positioning of micro-electrodes at the motor endplate of the costal diaphragm was confirmed at the beginning of an experiment by electrophysiological measures (i.e., rise-to-peak or 10–90% rise times of miniature endplate potentials (mEPPs) <2 ms) as well as by *post-hoc* fluorescently-labeled α-bungarotoxin labeling. Muscle action potentials (APs) were recorded after treatment with 50 μM BHC for 30 min, followed by 30 min of washing. EPPs were recorded after treatment with 2.3 μM μ-CTX. Signals were amplified using an Axoclamp 900A amplifier, digitized at 2 KHz using a Digidata 1550 and recorded using Axoscope software before being analyzed with the Clampfit data analysis module within pCLAMP10 software (Molecular Devices, Sunnyvale, CA, USA). mEPP, EPP, and AP decay times were calculated by measuring the descent time from peak to half amplitude, and halfwidths were calculated by measuring the time between half-maximal amplitudes on upslope and downslope. Only muscle fibers with resting membrane potentials between −60 and −75 mV were included for analysis. Tonic stimulation episodes of the phrenic nerve over 10 Hz were separated by 30-min rest periods to allow recovery. In order to calculate percent failure (APs) or percent transmitter release rundown (EPPs), the average of three potentials at each second of the 30-s train was taken and expressed as a percent of the first three potentials. For comparisons of APs to EPPs in response to 100 Hz (e.g., **Figure 2E**), the electrode was left in the same cell in between BHC and μ-CTX treatment. This experiment therefore produced only one recording in each drug per animal, and was performed four times with the same results.

### Tension recording of specific force

Longitudinal strips of diaphragm muscle containing the phrenic nerve were isolated by making two lateral cuts from the central tendon to the thoracic wall ~2–4 mm apart. The preparation was secured to the bottom of a Sylgard-lined dish using two sutures from the rib cage looping to hooks on the dish. A single fiber of braided suture silk (6–0, Ethicon, Sommerville, NJ, USA) was fastened around the central tendon and attached to a force transducer and amplifier (Transbridge 4 M, WPI, Sarasota, FL, USA). A suction electrode was attached to the phrenic nerve to provide suprathreshold nerve stimulation at 1, 10, 20, 40, and 100 Hz. Optimal muscle length and stimulation voltage were determined from micromanipulation of muscle length to produce peak force. Contractile activity was digitized using a Digidata 1332A recorded on a PC running Axoscope 10 (Molecular Devices). After contractile recordings, the muscle strip was trimmed from the tendon and rib cage, blotted on a Kim wipe and weighed on an analytical balance. Specific force was calculated by dividing recorded force (mN) by the Cross Sectional Area, where CSA, in mm^2^, = strip mass in mg/[(optimal muscle length in mm)*(L/Lo)*(1.06 mg/mm^3^)]. L/Lo is the fiber to muscle length ratio (1.0 for diaphragm; Brooks and Faulkner, 1988).

### Optical recording of fiber shortening

Brightfield and fluorescence recordings were performed on a Nikon Eclipse FN1 upright microscope using Nikon Plan Fluor 4x and 10x lenses (Nikon, USA). Image sequences were captured using an Andor Neo (Andor Technology, Belfast, UK) sCMOS camera and transferred to a Windows-based PC using Nikon NIS Elements 4.1 (Nikon, USA). Image sequences were recorded at 25 frames per second, processed as 8-bit intensity units, converted to multipage TIFF files and imported into and analyzed by custom software (Volumetry G8d; GWH; Hennig et al., 2002). To measure contraction (fiber shortening) from brightfield movies, two contrasted regions were identified. In most situations, these were blood vessels. To enhance the signal:noise ratio of the tracking regions, spatio-temporal maps (ST maps) were constructed with the axis of averaging perpendicular to the long axis of the muscle fibers (e.g., **Figure 5A**). After thresholding, the movement of the two regions was tracked in the ST maps using edge detection, and the distance was then calculated between them. In this manner, the shortening between the regions could be calculated regardless of the overall displacement of the diaphragm during stimulation.

### Calcium imaging

The diaphragm of CAGGS-GCaMP3 mice was excited at 470 nm by a Lumencor Spectra X light engine (Lumencor, Beaverton, Oregon, USA). Image sequences were captured, recorded and analyzed as described above. In order to calculate maximal fluorescence (Fmax) exhibited by GCaMP3 in skeletal muscle, 0.5 M potassium chloride (KCl) was added to diaphragm preparations, as larger fluorescences were not observed in response to treatment with ionomycin (to allow influx of extracellular calcium) or to the RyR agonist caffeine or the SERCA antagonist cyclopiazonic acid (to deplete sarcoplasmic reticular calcium stores). First, the optimal dynamic range of the camera sensor was established in response to KCl. Although the Andor Neo cMOS chip sensor reportedly exhibits saturation in 16-bit mode at 65,000 counts (2e16), by pseudocoloring pixel saturation, GCaMP3-expressing muscle tissue exhibited saturation at 20X magnification and 3 × 3 binning when the brightness bar on the lookup table was moved to the left of 2000 counts. Therefore, all nerve stimulation experiments were carried out with this bar set to 110% of this level, or ~2200 counts. When nerve stimulation experiments were performed in this context, the largest fluorescence (the initial peak in response to 20–100 Hz stimulation), was never higher than 500 counts, confirming that neither the camera sensor nor the biosensor were saturated. A number of spatio-temporal maps (ST maps) were used to portray and analyze the pattern of calcium transients in muscle cells in response to nerve stimulation. The main type was the standard deviation map, which was generated by calculating the standard deviation of fluorescence intensity at every pixel immediately prior to the application of the stimulus (0.5–1.0 s) extending to 30–50 s depending on the stimulus used. Standard deviations were color coded (“Fire” CLUT) to more accurately portray the relative intensity of the signal in muscle cells in relation to background noise (Hennig et al., 2015). To better appreciate the heterogeneity of muscle fiber responses during different stimulation paradigms, ST maps were colored and overlaid and summary images were created using different statistical procedures including average intensity and the standard deviation of intensity (e.g., **Figures 6B,C**). The change in intensity was referenced to baseline or maximal intensity (typically 1 s after phrenic nerve stimulation).

### Statistics

All data were expressed as means ± *SD* Student's *t*-tests assuming equal variance, with or without the Bonferroni correction, were used to assess differences between two samples. A *P* < 0.05 was considered significant.

## Results

### Electrophysiological characterization of the effect of BTS as well as the hydroxycoumarin derivative, BHC, on muscle responses to nerve stimulation

The effect of BTS on nerve-evoked adult mouse diaphragm contractions was first examined. Treatment with 1 μM BTS failed to reduce nerve stimulation-induced muscle contractions, but 5 μM BTS completely inhibited them to a point at which they were unresolvable in bright-field videos (data not shown). At this concentration, muscle action potentials (APs) in response to nerve stimulation were much smaller than expected (10–20 mV vs. 70–90 mV; van Lunteren and Moyer, 1998; *n* = 5 from three mice; data not shown). At higher doses of BTS, such as those used in previous studies of the toad NMJ (50 μM; Etherington et al., 2014), nerve-evoked muscle potentials decreased below 5 mV (*n* = 5 from three mice; Supplementary Figure 1).

The effects on nerve-evoked muscle contraction of the two hydroxycoumarin derivatives that also blocked myosin ATPase activity in purified muscle cells (BHC and 3-(N-butylethanimidoyl)-4-hydroxy-6-nitro-2H-chromen-2-one; Cheung et al., 2002) were then assessed. Only BHC reduced muscle contractile responses to a level that was unresolvable (data not shown). At a dose of 10 μM, BHC partially blocked muscle contractions, but at 50 μM, BHC reduced diaphragm muscle movement to a level that was unresolvable across a wide range of phrenic nerve stimulation frequencies (see below, **Figure 2**). Complete paralysis was maintained for over 3 h after washout before movement gradually returned (*n* = 3 from three mice; data not shown).

**Figure 1:**
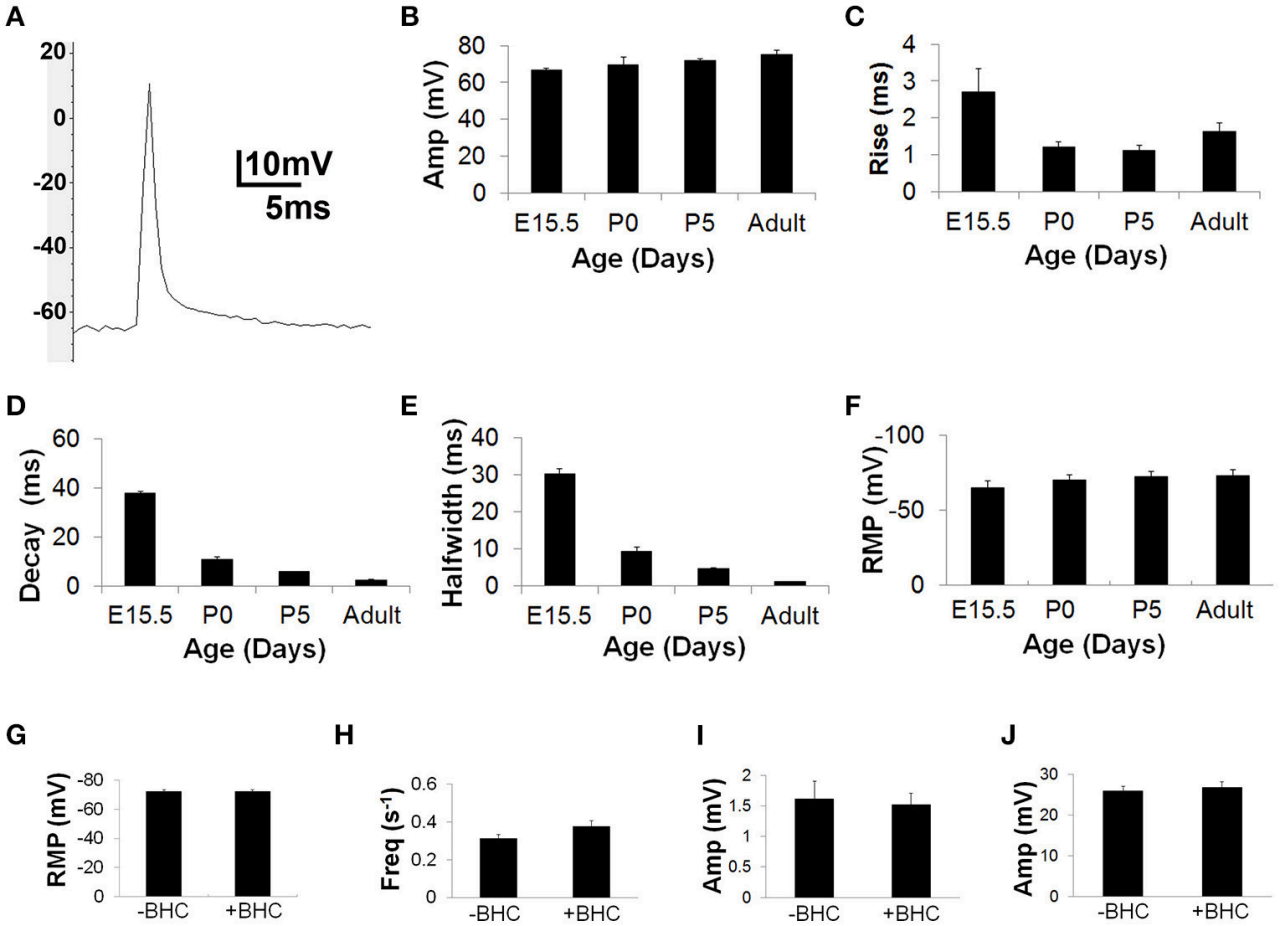
**BHC permits the measurement of nerve-evoked muscle action potentials (APs). (A)** Representative image of an AP recorded in the presence of 50 μM BHC with a sharp intracellular electrode from the motor endplate of an adult mouse diaphragm muscle fiber in response to a single suprathreshold square wave phrenic nerve impulse. Note overshoot. Average amplitudes are lower (E15.5 AP amp = 65.5 ± 1.6 vs. adult AP amp = 75.3 ± 2.2 mV; *P* < 0.001; *n* = 55 from 10 mice; **B**), and 10–90% rise-to-peak (E15.5 R2P = 2.7 ± 0.7 vs. adult R2P = 1.6 ± 0.2 ms; *P* < 0.001; *n* = 55 from 10 mice; **C**), 100–50% decay (E15.5 T2D = 37.8 ± 1.6 vs. adult T2D = 2.4 ± 0.2 ms; *P* < 0.001; *n* = 55 from 10 mice; **D**), and 50–50% halfwidth (E15.5 HW = 30.2 ± 2.7 vs. adult HW = 1.2 ± 0.1 ms; *P* < 0.001; *n* = 55 from 10 mice; **E**) of muscle APs are significantly longer at E15.5 (E15.5) than postnatal stages. **(F)** Average resting membrane potential (RMP) is less negative at E15.5 vs. postnatal ages (E15.5 RMP = −64.8 ± 4.3 vs. adult RMP = −70 ± 2.6 mV; *P* < 0.05; *n* = 5 from three mice; **F**). The RMP is similar in the presence or absence of 10 μm BHC in the adult; (−72.4 ± 2.3 vs. −72.2 ± 3.6 mV; *P* = 0.46; *n* = 5 from three mice; **G**). The frequency of miniature endplate potentials (mEPPs; 0.27 ± 0.04 vs. 0.38 ± 0.03 mEPPs/s; *P* = 0.06; 10 s analyzed; *n* = 8 from five mice; **H**), as well as the amplitude of mEPPs (1.6 ± 0.3 vs. 1.5 ± 0.2 mV; *P* = 0.1; *n* = 22 from five mice; **I**) and EPPs (26.2 ± 1.1 vs. 26.7 ± 1.3 mV; *P* = 0.06; *n* = 22 from five mice; **J**) are similar in the presence or absence of BHC in the adult. All values expressed in means ±*SD*.

In contrast to BTS, BHC treatment failed to attenuate the size of nerve-evoked muscle APs, which exhibited parameters similar to published estimates obtained in response to single pulses of direct muscle stimulation of adult rat diaphragm (Adult AP amplitude = 75.3 ± 2.2 mV; *n* = 55 from 10 mice; vs. 75.6 ± 3.2 mV; Delbono and Kotsias, 1987). To determine the effects of age, nerve-evoked muscle APs were recorded from diaphragm muscle derived from embryonic day 15.5 (E15.5), postnatal day 0 (P0) and P5 as well as adult mice. APs were lower in amplitude and longer in duration during embryonic periods but became significantly larger and shorter in duration immediately after birth (Figures 1A–F). Finally, to test whether BHC exerted effects on neuromuscular physiology other than blocking contractility, resting membrane potential, miniature endplate potential (mEPP) frequency and amplitude, and EPP amplitude were all measured in the presence or absence of BHC (Figures 1G–J). No significant differences in any of these measures were observed, suggesting that BHC does not affect the ion dynamics underlying muscle APs.

### Neural transmission failure (NTF) in response to high-frequency nerve stimulation

**Figure 2:**
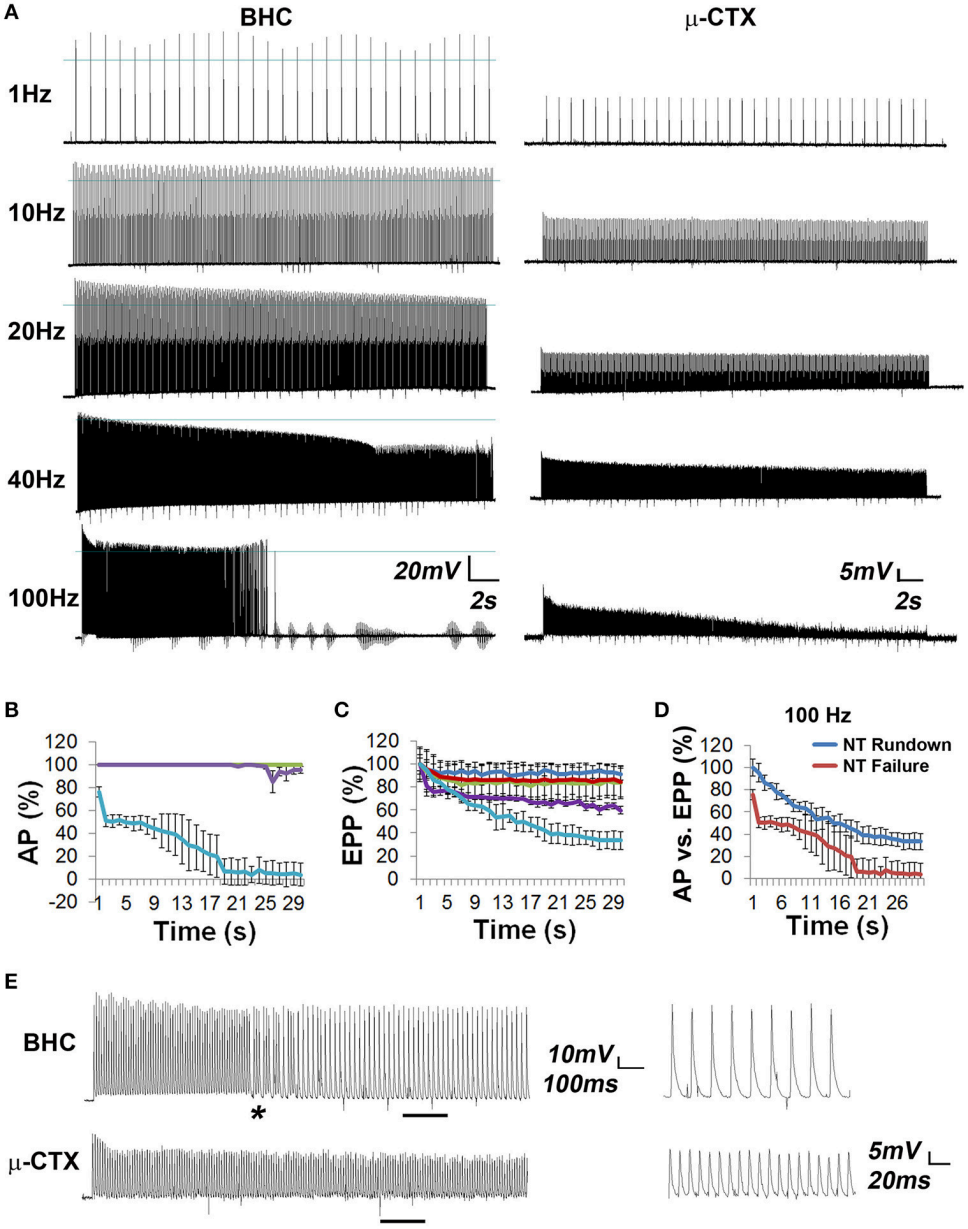
**Comparison of EPPs vs. muscle APs in whole diaphragm in response to high-frequency nerve stimulation (HFS) shows that muscle APs fail intermittently, whereas neurotransmitter release falls gradually. (A)** Left panels show representative muscle AP traces, recorded in the presence of 50 μM BHC, in response to 30-s trains of tonic phrenic nerve stimulation at different frequencies. Overshoots are portions of the muscle AP above 0 mV, which is demarcated by a blue line in each trace. Right panels show representative EPP traces, recorded in the presence of 2.3 μM μ-CTX, in response to the same stimuli. Note the presence of muscle AP failure in response to 100 Hz. **(B)** The percent of successfully transmitted muscle APs per second (AP %), over time, in response to different frequencies of HFS (blue = 1 Hz; red = 10 Hz; green = 20 Hz; purple = 40 Hz; light blue = 100 Hz. The straight line at 100% for 1, 10, and 20 Hz reflects the lack of failure in response to 30 s of stimulation at these frequencies. **(C)** Synaptic rundown of released neurotransmitter (NT), measured by EPP amplitude, in response to the same trains of stimuli. **(D)** Comparison of NT release rundown (in presence of μ-CTX) and neural transmission failure (NTF) in presence of BHC. **(E)** Left panel shows a comparison of the first few seconds of AP (BHC) and EPP (μ-CTX) traces in response to 100 Hz stimulation. Asterisk represents first failed AP. Underlined areas are enlarged in right panel and show the pattern of APs and EPPs after a corresponding period of HFS. For every muscle AP, there are two, nearly equal-sized EPPs.

**Figure 3:**
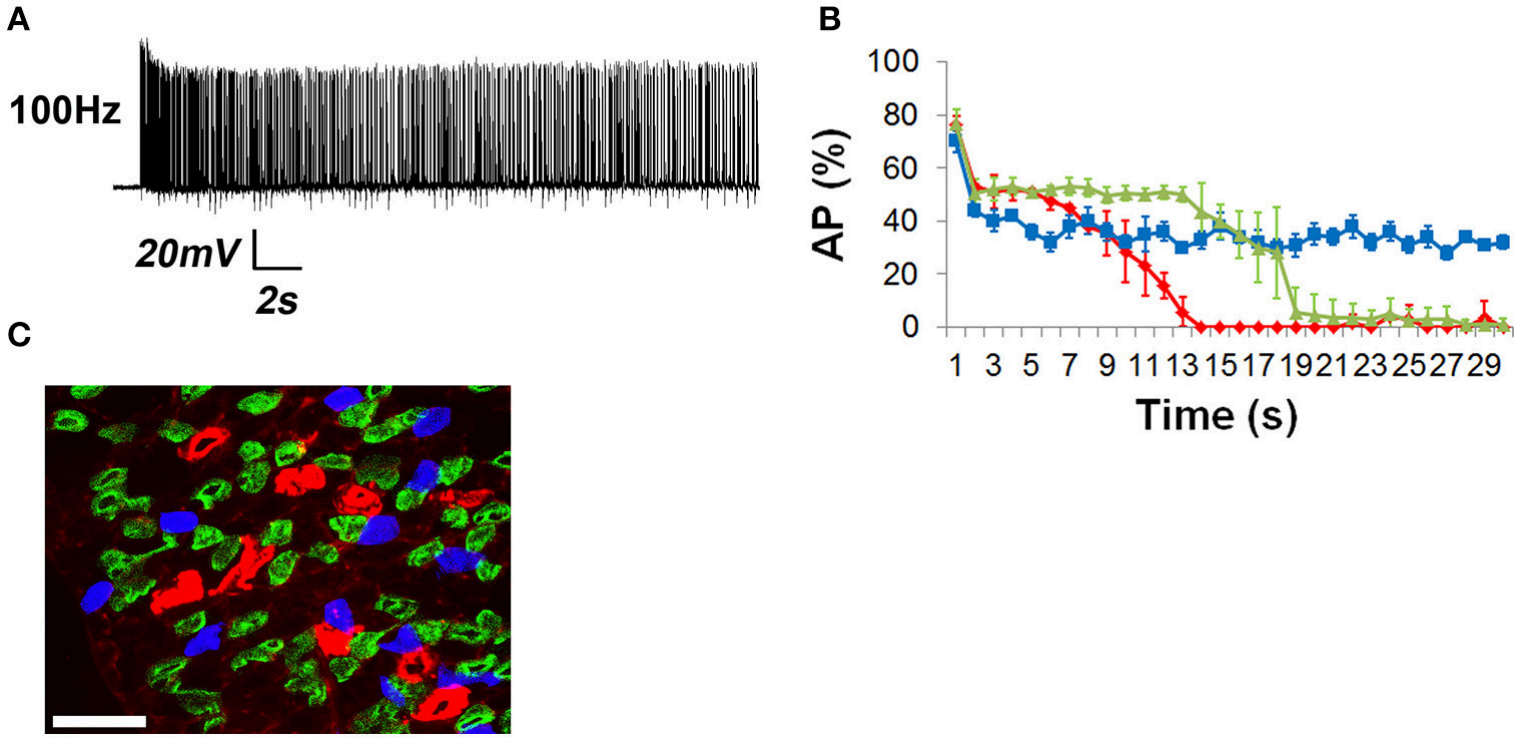
**The percentage of muscle APs with defined times to neural transmission failure coincides with the percentage of muscle fibers expressing fatigue-conferring myosin isoforms. (A)** Representative trace of a muscle fiber exhibiting only intermittent failure for the entire 30-s period in response to 100 Hz stimulation. Note the difference between this cell and that in Figure 2A. **(B)** When cells were plotted individually according to when they exhibited NTF, three subpopulations emerged, those exhibiting failure near 10 s (blue), those near 20 s (green), and those only displaying partial failure even after 30 s, similar to the trace in **(A)**; (blue line). **(C)** Fresh-frozen cross sections of muscle stained with antibodies recognizing Type I, slow, oxidative MHC (blue), Type IIA fast fatigue-resistant, glycolytic/oxidative MHC (green), and Type IIB fast-fatiguable glycolytic MHC (red), or no antibody labeling (Type IIx). Scalebar = 100 μm. The relative percentage of these myosin-expressing subpopulations is similar to the relative percentage of muscle subpopulations fatiguing in response to 100 Hz at different times, as shown in **(B)**.

High-frequency nerve stimulation (HFS) was used to induce neurotransmission failure in the adult mouse diaphragm. Although phrenic MNs discharge phasically (e.g., duty cycle of 0.33) during respiration, they also produce tonic contraction of the diaphragm to elevate intra-abdominal pressure during expulsive maneuvers such as vomiting, coughing or defecation, to aid in postural control during upper limb movement (Hodges and Gandevia, 2000), or to maintain head-level arterial pressure during the production of anti-gravity straining maneuvers by military pilots (Bain et al., 1997). Tonic stimulation of the phrenic nerve also results in accelerated depletion of neurotransmitter compared to phasic activation (Moyer and van Lunteren, 1999), facilitating the study of fatigue. At tonic frequencies <40 Hz, muscle APs were reliably obtained in response to every pulse of nerve stimulation for up to 30 s (Figures 2A,B). At 40 Hz, nerve stimulations failed to produce muscle APs between 20 and 30 s (Figures 2A,B). At 100 Hz, these failures were first observed toward the end of the initial second of stimulation, with the percentage of failed muscle APs increasing dramatically over time (Figures 2A,B). Muscle fiber response to 100 Hz nerve stimulation could be grouped into one of three subtypes: those completely failing to produce nerve-evoked muscle APs after 10–15 s, those completely failing to generate APs after 20 s, and those exhibiting intermittent but not complete AP failure (Figures 3A,B). The proportion of muscle fibers in each failure category was then correlated to the proportion of different fiber subtypes based on MHC isoform composition, which defines these subtypes according to fatiguability. The relative percentage of muscle fibers displaying complete transmission failure most quickly (18 ± 5.2%, *n* = 15 from seven mice) was similar to that exhibiting immunoreactivity to the MHC subtype associated with fast-fatiguable, glycolytic Type IIB muscle fibers (13.0 ± 2.3%; *n* = 10 from three mice; Figure 3C). Similarly, the percentage of fibers exhibiting failure slightly later (66.3 ± 12.1%; *n* = 15 from seven mice) was similar to that exhibiting expression of MHC Type IIA and Type IIX fast fatigue-resistant, oxidative/glycolytic myosin (71.4 ± 4.0%; *n* = 10 from three mice). Finally, the percentage of fibers exhibiting intermittent APs even after 30 s of 100 Hz nerve stimulation (15.7 ± 0.7%; *n* = 15 from seven mice) was similar to that expressing MHC Type I slow oxidative myosin (13.5 ± 2.7%; *n* = 10 from three mice). The relative percentages of these MHC-immunoreactive muscle fiber subtypes in the adult mouse diaphragm were similar to those of previous reports (Agbulut et al., 2003; Fajardo et al., 2016). Together, these results show that BHC permits the direct measurement of NTF at the vertebrate NMJ.

In order to examine if reduced acetylcholine (ACh) release contributed to NTF, intracellular muscle fiber potentials were recorded in response to the same series of frequencies of phrenic nerve stimulation in the presence of μ-CTX, which blocks Na_v_1.4 channels and thus permits the measurement of the ACh-mediated EPP. The synaptic rundown of ACh release, measured as EPP amplitudes, was then correlated to muscle AP failure rates. Stimulation at 1, 10, and 20 Hz generated a modest and progressive reduction of EPP amplitude after 30 s of stimulation, with the majority of this decrease occurring in the first second, whereas 40 and 100 Hz stimulation produced this initial decrease followed by an additional prolonged reduction of EPP height (Figures 2A,C). The change from initial to final EPP amplitude of 28.0 ± 3.87 mV to 23.4 ± 3.58 mV (*n* = 8 from four mice) after 30 s of stimulation at 20 Hz in the presence of μ-CTX is insufficient to cause NTF, because 20 Hz stimulation in the presence of BHC resulted in 100% successful APs. These results are consistent with the lowest previously reported safety factor in rat diaphragm muscle, 1.7, which would require a drop in EPP amplitude from 28 to 17.6 mV before causing NTF failure (Wareham et al., 1994). In contrast, the change of initial to final EPP amplitude in response to 30 s of 40 Hz stimulation (28.0 ± 1.39 mV to 16.2 ± 0.82 mV; *n* = 9 from four mice) is below this estimate of the safety factor (1.73-fold reduction), consistent with the observation of a small percentage of failed muscle APs in response to 40 Hz nerve stimulation in the presence of BHC.

Although a 1.7-fold reduction of EPP amplitude resulted in NTF in response to 40 Hz stimulation, it does not produce complete NTF. Rather, only a small percentage of neural APs failed to produce muscle APs as EPP amplitude fell below this threshold. In order to investigate this further, EPPs and APs were examined in response to 100 Hz stimulation, which produced complete NTF in some muscle fibers (Figure 2A). The relative percentage of successfully transmitted APs changed over time. Initially, failed muscle APs occurred within the first second. In the next 4–5 s, every other muscle AP failed. During this time, EPPs not only did not fall below the safety factor, they failed to change significantly in amplitude in an every-other pattern (Figures 2D,E). Between 10 and 15 s after the onset of 100 Hz stimulation, there was a marked decrease in the percentage of successfully transmitted muscle APs. This decline is correlated with the reduction of EPP amplitudes below the safety factor. Finally, after 15 s, the percentage of successful muscle APs dropped again, coinciding with a further reduction of EPP amplitudes. Together, these results provide evidence that although percentage rates of NTF are largely correlated to levels of neurotransmitter release rundown, impairments of neurotransmission occur intermittently, and in some cases, independently of reduced neurotransmitter release.

### Relationship of NTF to muscle fatigue (isolated strips)

**Figure 4:**
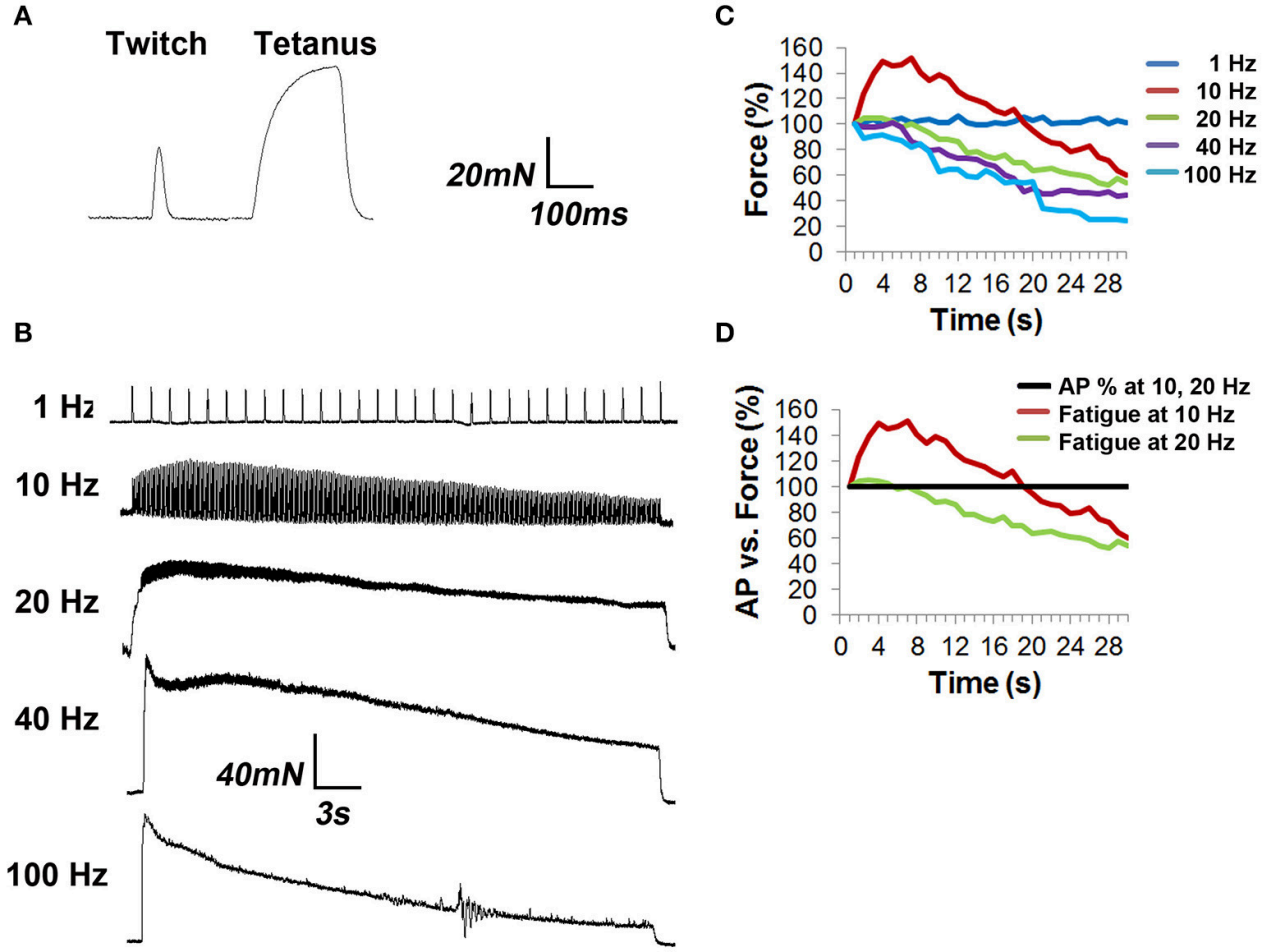
**Tension studies in diaphragm strips show fatigue in response to frequencies of nerve stimulation that fail to show neural transmission failure. (A)** Diaphragm strips with intact phrenic nerve were excited with single suprathreshold square wave nerve pulses (twitch) or 330 ms of nerve stimulation at 70 Hz (tetanus). **(B)** Representative traces of diaphragm strip responses to 30 s trains of tonic nerve stimulation at different frequencies. Note the several-second buildup to peak tension in response to 10 Hz stimulation, followed by fatigue, as well as the progressively enhanced fatigue in response to 20, 40, and 100 Hz stimulation. **(C)** The percent specific force (Force %), over time, in response to different frequencies of HFS, shows that all frequencies above 1 Hz produce fatigue. Error bars left off for clarity. **(D)** Comparison of force to AP success rate in response to 10 and 20 Hz shows that peak tension declines in the absence of NTF at these frequencies.

In order to assess the effects of NTF on muscle fatigue, tension produced by 1–100 Hz phrenic nerve stimulation was measured in isolated diaphragm strips. Muscle fatigue, or the reduction of muscle force that occurs in response to repeated stimulation, is potentially caused by a variety of mechanisms affecting the central drive to MNs, neurotransmission, and the response of the muscle fiber to neurotransmission (Sieck and Prakash, 1995). A 330 ms pulse of 70 Hz stimulation, which produces a fused tetanus, elicited a specific force of 136.9 ± 12.2 mN/mm^2^ (*n* = 3 from three mice; Figure 4A), similar to previous reports (Fajardo et al., 2016). Continued phasic stimulation of the phrenic nerve at this frequency (duty cycle 0.33) produced fatigue (72 ± 3 stimuli to achieve 60% peak force, which was achieved on the second 330 ms stimulus).

In order to measure the effect of tonic nerve stimulation frequency on muscle fatigue, the difference between ending and peak forces was measured. In response to 1 Hz for 30 s, which fails to produce NTF, specific force was unchanged (peak vs. ending forces of 59.6 ± 4.2 vs. 60.3 ± 3.3 mN/mm^2^, *P* = 0.39; *n* = 3 from three mice; Figure 4B). However, in response to 10 and 20 Hz, which also fails to produce NTF, after a several-second rise to peak tension, the force declined considerably (10 Hz; peak vs. ending forces of 83.2 ± 7.7 vs. 33.2 ± 3.6 mN/mm^2^; 60% decline; *P* < 0.05; *n* = 6 from three mice; 20 Hz; peak vs. ending forces of 110.2 ± 9.3 vs. 57.2 ± 6.1 mN/mm^2^; 48% decline; *P* < 0.05; *n* = 6 from three mice; Figures 4B–D). At higher frequencies that caused NTF, further reductions of tension were observed (40 Hz; peak vs. ending forces of 114.2 ± 14.2 vs. 50.6 ± 16.6 mN/mm^2^; 56% decline; *P* < 0.005; *n* = 3 from three mice; 100 Hz; peak vs. ending forces of 98.4 ± 17.4 vs. 24.5 ± 6.1 mN/mm^2^; 75% decline; *P* < 0.005; *n* = 3 from three mice; Figure 4C). Total force, measured as the area under the 30-s curve (AUC), was 1762 ± 145 mN/mm^2^.s in response to 10 Hz, reached its maximum at 20 Hz (2379 ± 176 mN/mm^2^.s), was unchanged at 40 Hz (2238 ± 107 mN/mm^2^.s), and declined at 100 Hz (1650 ± 181 mN/mm^2^.s; *n* = 3 from three mice). Therefore, despite the observation that neither 10 Hz nor 20 Hz nerve stimulation produce NTF, muscle fatigue is observed, suggesting that fatigue in response to these frequencies of HFS is mediated by factors downstream of neurotransmission.

### Relationship of NTF to muscle fatigue (intact diaphragm)

**Figure 5:**
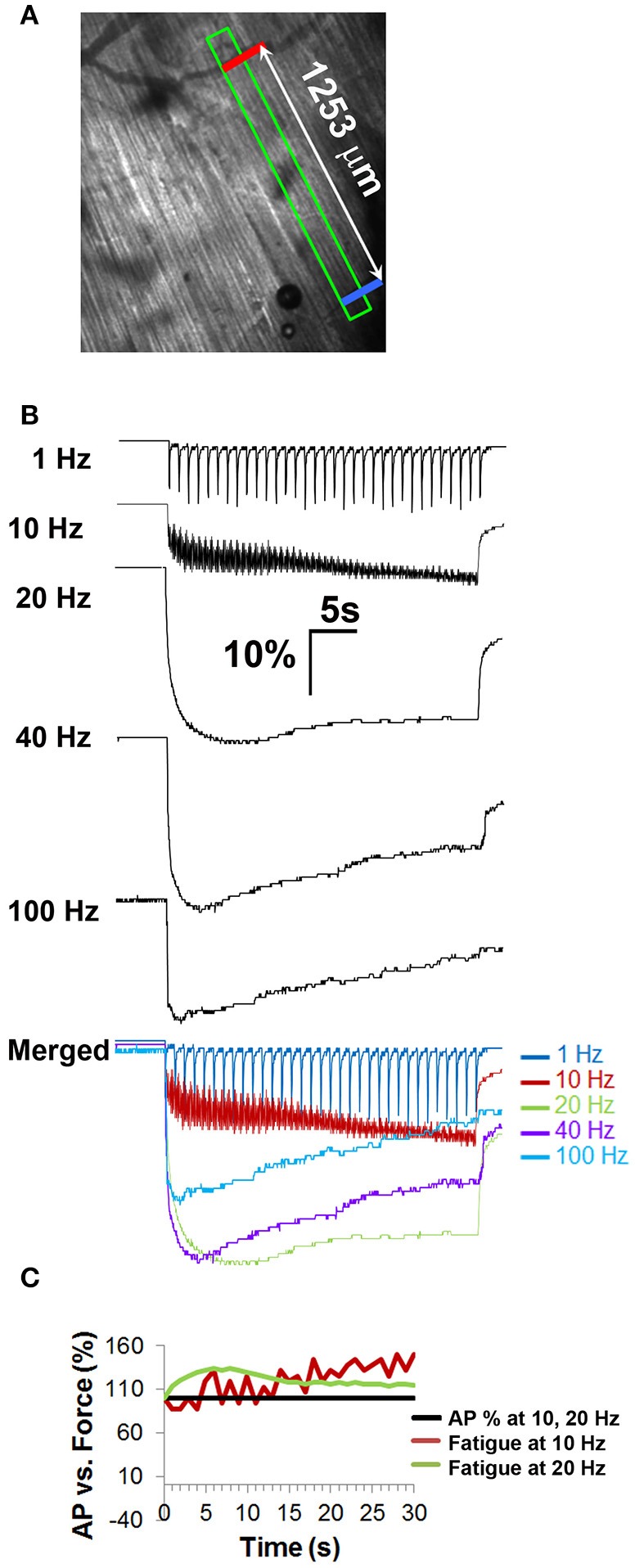
**Fiber shortening studies in whole diaphragm fail to show fatigue in response to frequencies of nerve stimulation that fail to show neural transmission failure. (A)** Field of view from which optical measurements were taken and distance between the two contrasted regions (indicated by red, blue bars) that were tracked in response to nerve stimulation. **(B)** Representative percent control traces of fiber shortening in response to different frequencies of tonic nerve stimulation. Note the change to tetanus in response to 10 Hz. Note also the lack of fatigue in response to 10 and 20 Hz stimulation, in contrast to tension studies. **(C)** Comparison of force to AP success rate in response to 10 and 20 Hz shows that peak fiber shortening does not decline in the absence of NTF at these frequencies.

Because electrophysiological measurements were obtained in whole diaphragms, whereas those of tension were produced from diaphragm muscle strips, the comparison between data generated by these techniques may not be completely valid. For example, force decline in strips may be enhanced by the absence of forces acting transversely against the length of the muscle fiber (Margulies et al., 1994). To better match muscle responses and electrophysiological measurements, muscle shortening was measured optically between two fixed regions in response to nerve stimulation in bright-field videos of whole diaphragms; (Figure 5A; Video 1). The maximal rising slope of fiber shortening increased proportionally with nerve stimulation frequency (3668 ± 1166 μm/s for 40 Hz vs. 5610 ± 999 μm/s for 100 Hz; *P* < 0.05, *n* = 3 from three mice; for example; compare rising slopes in merged image in Figure 5B). Similar to results of tension studies, the highest total shortening (AUC) obtained by optical methods was observed in response to 20 Hz nerve stimulation, followed by 40 Hz and then 100 Hz stimulation (1 Hz = 531 ± 52 μm.s; 10 Hz = 3386 ± 278 μm.s; 20 Hz = 8835 ± 1176 μm.s; 40 Hz = 7485 ± 624 μm.s; 100 Hz = 4928 ± 762 μm.s; *n* = 3 from three mice). To assess fatigue, the difference between peak and length at the end of stimulation (ending length) was measured. Similar to that measured by tension recording, muscle shortening measured by optical methods failed to exhibit fatigue in response to 1 Hz stimulation (peak vs. ending length changes of 96.5 ± 6.8 μm vs. 102.7 ± 11.3 μm; *P* = 0.102; *n* = 4 from three mice; Figure 5B). However, in contrast to tension measurements with diaphragm strips, this technique revealed that 10 Hz nerve stimulation failed to cause fatigue in whole diaphragms. In fact, ending length was essentially equal to peak length (156.3 ± 26 μm). Interestingly, whereas diaphragm strips failed to exhibit fusion of muscle twitches into tetanus in response to 10 Hz nerve stimulation, whole diaphragm measured optically exhibited a transition from twitch to tetanus in response to 10 Hz (Figures 4B, 5B). Similarly, although the peak length change induced by 20 Hz nerve stimulation appeared unable to be maintained, this difference was not statistically significant (peak vs. ending length changes after 20 Hz were 339.6 ± 34.3 vs. 293.2 ± 42.1 μm; *P* = 0.053; *n* = 4 from three mice). In contrast, 40 and 100 Hz nerve stimulation produced fatigue (Figure 5B). However, in both cases, similar to the effects of 10 and 20 Hz nerve stimulation, fatigue produced by 40 and 100 Hz stimulation was smaller with optical vs. tension measurements. For example, peak vs. ending length changes after 40 Hz were 335.8 ± 83.8 vs. 214 ± 56.9 μm; *P* < 0.05; *n* = 4 from three mice (36.1 vs. 56%); and after 100 Hz were 239.3 ± 31.7 vs. 114 ± 23.2 μm; *P* < 0.005; *n* = 4 from three mice (52.3 vs. 75%). Therefore, in contrast to tension recording of diaphragm strips, optical recording of fiber shortening in whole diaphragms fails to show fatigue in response to 10 and 20 Hz nerve stimulation (Figure 5C).

### Relationship of NTF, muscle fatigue, and cytosolic calcium levels

**Figure 6:**
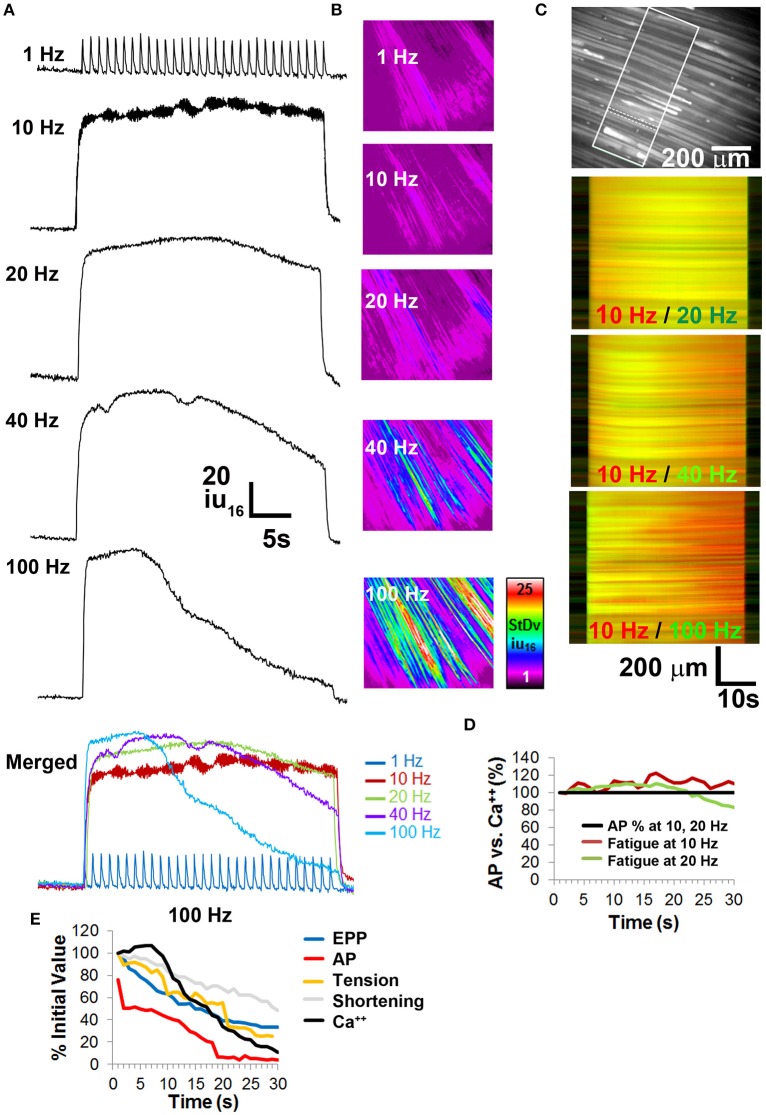
**Calcium imaging studies in whole diaphragm show no fatigue in response to frequencies of nerve stimulation that show no neural transmission failure. (A)** Representative standard deviation (SD) of calcium intensity changes (16-bit intensity units; iu_16_) within BHC-treated muscle fibers from the diaphragm of CAGGS-GCaMP3 mice in response to different frequencies of tonic nerve stimulation. **(B)** Spatio-temporal (ST) maps of the standard deviation (*SD*) of intensity represent the loss of intensity over time as a signal that increases with nerve stimulation frequency. **(C)** Top image illustrates the region of the costal diaphragm, the dotted lines surround a muscle fiber from which the intensity changes in **(A)** were generated. Boxed region shows population of muscle fibers whose intensities signals were tracked over time for SD maps in **(B)** or intensity map subtractions in (lower three images in **C**). Lower images represent differential ST maps of calcium intensity over time in response to different frequencies of nerve stimulation. In the 10/20 Hz comparison, the yellow signal from left to right indicates that the 10 and 20 Hz ST intensity maps (red, green) are equally maintained over the 30 s duration, whereas the red signal in the bottom two comparisons indicates a loss of signal in the green 40 or 100 Hz ST intensity maps over time. **(D)** No significant loss of calcium signal is observed over time at frequencies that fail to induce NTF. **(E)** Comparison of EPP rundown, AP transmission success rate, muscle force via tension or shortening, and calcium intensity, in response to 100 Hz nerve stimulation.

Calcium release from the sarcoplasmic reticulum into the cytosol transduces the nerve-induced muscle AP into a contractile response. Fatiguing tetanic stimulation of isolated muscle fibers causes a reduction of calcium release (Allen et al., 2008). In order to determine whether calcium release was differentially affected in response to muscle fatigue-inducing nerve stimulation, whole diaphragms of transgenic mice expressing the genetically encoded calcium indicator GCaMP3 (CAGGS-GCaMP3) were treated with BHC to block movement and imaged in response to 30-s trains of phrenic nerve stimulation. Similar to the production of muscle APs, 1 Hz nerve stimulation reliably produced calcium transients for 30 s (Figure 6A). Similar to fiber shortening, the maximal rising slope of calcium-induced fluorescence increased proportionally with nerve stimulation frequency (compare in merged image in Figure 6A). Also similar to fiber shortening, the highest total transient intensity (AUC) was observed in response to 20 Hz nerve stimulation, followed by 40 Hz stimulation (1 Hz = 2527 ± 721 iu_16_.s; 10 Hz = 4108 ± 1230 iu_16_.s; 20 Hz = 4258 ± 640 iu_16_.s; 40 Hz = 4214 ± 1327 iu_16_.s; 100 Hz = 3640 ± 591 iu_16_.s; *n* = 5 from five mice Figure 6A). These responses to nerve stimulation were submaximal, because bath application of either 0.5 M potassium chloride or 100 μM carbachol elicited calcium transients with higher amplitudes (data not shown).

To examine calcium responses during fatigue, peak intensity and the intensity at the end of stimulation (ending transient intensity) were analyzed. In response to 30 s of 1 Hz nerve stimulation, calcium transient intensity exhibited no significant change in amplitude, similar to the effects of this stimulus on EPPs, APs, tension, and shortening (peak intensity of 18.4 ± 4.4 SD iu_16_ vs. end intensity of 16 ± 3.8 SD iu_16_; *P* = 0.19; *n* = 5 from three mice; Figure 6A; Video 2). Although the temporal dynamics of GCaMP3 preclude tracking all components of individual transients at frequencies >6 Hz (Tian et al., 2009), the overall change in intensity in response to high-frequency trains can be measured. Similar to the fiber shortening studies, transient intensity failed to decrease significantly in response to 30 s of 10 or 20 Hz stimulation (10 Hz peak and ending intensities were 76.3 ± 9.3 vs. 70.3 ± 10.3 SD iu_16_; *P* = 0.17; 20 Hz peak and ending intensities were 83.6 ± 19 vs. 64.2 ± 22.6 SD iu_16_; *P* = 0.051; *n* = 5 from three mice Figures 6A–D). In contrast to 10 and 20 Hz nerve stimulation, 40 and 100 Hz caused a profound loss of transient intensity, similar to the loss of peak length changes (40 Hz peak and ending intensities were 88.2 ± 21.3 vs. 41.2 ± 14.6 SD iu_16_; *P* < 0.005; 100 Hz peak and ending intensities were 88.8 ± 10.5 vs. 8.2 ± 5.3 SD iu_16_; *P* < 0.001; *n* = 5 from three mice). The loss of calcium transient intensity in response to 40 and 100 Hz stimulation was also represented by spatio-temporal (ST) maps of standard deviation, in which the change in intensity values over time in a population of fibers is represented by differences of color intensity within them (Figure 6B). By superimposing pseudo-colored ST maps of calcium transients at fatiguing vs. non-fatiguing frequencies, both the spatial and temporal characteristics of changes in calcium transient intensities could be portrayed. For example, merged ST maps of 10 Hz (red) and 20 Hz (green) stimulations appear predominantly yellow throughout the 30 s period, representing similar intensities over time throughout the recorded area of the diaphragm. Merged ST maps of 10 Hz (red) and either 40 or 100 Hz (green) show a change from yellow to red over time, with the loss of green signal representing the loss of transient intensity in response to these higher frequencies (Figure 6C). The loss of intensity was not uniform throughout the diaphragm, with some regions showing much earlier declines than other, often adjacent regions. Interestingly, in response to fatiguing stimulation, the flashing of individual muscle fibers could be observed (early portion of 100 Hz video, Video 2) consistent with the pattern of intermittent failure observed with electrophysiological recordings. Together, these results demonstrate the relationship between calcium responses in adult muscle fibers and fatiguing nerve stimulation, illustrating the utility of BHC for measuring calcium dynamics within populations of the same fibers over time in *ex vivo* neuromuscular preparations.

### Evaluation of NTF and fatigue in embryonic wild-type and Vamp2 mutant mice

We tested whether BHC could permit the study of neurotransmission and fatigue at E15.5, shortly after branches of the phrenic nerve terminal navigate to and contact the pre-patterned AChR cluster-enriched motor endplate. At these stages, although the mRNA for μ-CTX-resistant Na_v_1.5 channels predominates over μ-CTX-sensitive Na_*v*_1.4 channels (Lupa et al., 1993; Wilson et al., 2011), the sodium currents underlying embryonic muscle APs have not been examined. Therefore, we investigated the effects of μ-CTX in E15.5 NMJs. Treatment with any dose failed to prevent nerve-evoked muscle APs (data not shown), consistent with a lack of functional Na_v_1.4 channel expression. However, in the presence of BHC, nerve stimulation produced measurable muscle APs (Figures 1B–F). The rise-time of these APs was significantly longer than those from postnatal muscle, consistent with the possibility that NMJs at these early ages express slow, μ-CTX-resistant Na_v_1.5 channels (Wang et al., 1996).

**Figure 7:**
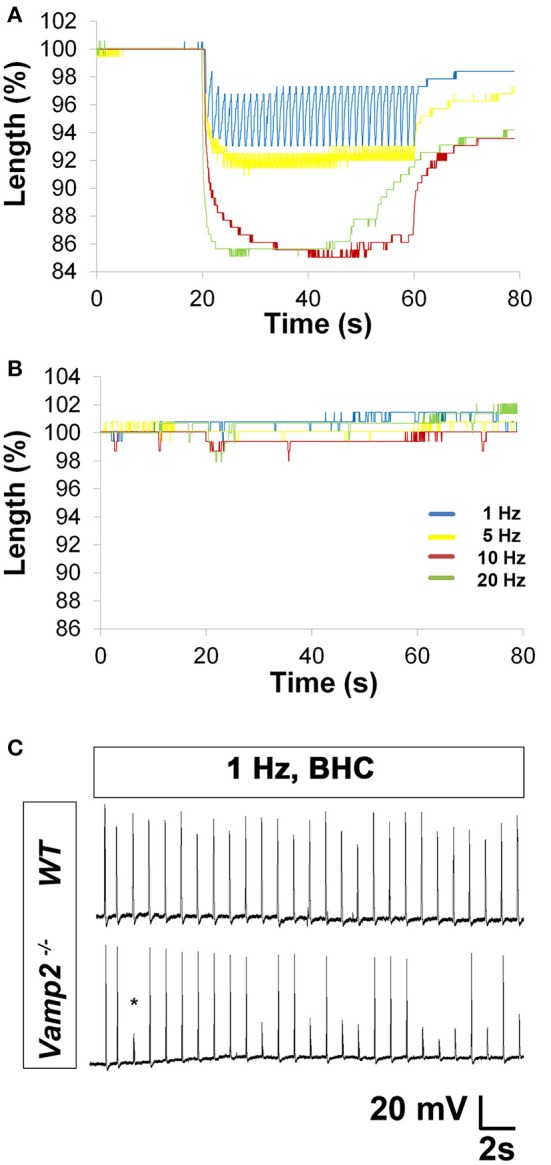
**Characterization of HFS in embryonic wild-type (WT) and ***Vamp2*** mutant mice. (A)** Representative merged percent control traces of fiber shortening in E15.5 WT mice. Note the progressive change in length (shortening) of embryonic muscle fibers in response to increasing nerve stimulation frequency. Note also the loss in maintenance of peak length change in response to 20 Hz (green). **(B)** In contrast, HFS produces profoundly less fiber shortening in E15.5 *Vamp2* mutants. Images to the right of each graph in **(A,B)** show the field of view from which data was obtained, including the distance between the two contrasted regions (indicated by red, blue bars) that were tracked in response to nerve stimulation. **(C)** Representative recordings from the diaphragms of E15.5 WT (top trace) or *Vamp2* mutant (bottom trace) in the presence of BHC show that although nerve stimulation can produce muscle APs in *Vamp2* mutants, even low frequencies result in NTF, illustrated by subthreshold muscle potentials (asterisk).

Embryonic *Vamp2* mutant mice exhibit profoundly impaired evoked release of neurotransmitter in the CNS and die shortly after birth (Schoch et al., 2001). In order to identify the frequency of nerve stimulation that was sufficient to produce fatigue in embryonic muscle, the phrenic nerve of E15.5 WT mice was tonically stimulated for 30 s at 1, 5, 10, and 20 Hz (Video 3), and diaphragm muscle fiber length changes were measured optically. Although 30 s of 1 Hz nerve stimulation failed to produce fatigue, the inability to maintain peak fiber shortening was observed toward the end of the 20 Hz stimulation period (peak vs. ending length changes of 160.3 ± 25 vs. 91.5 ± 20.1 μm; 42% decline; *P* < 0.005; *n* = 4 from three mice; Figure 7A). Fatigue was also examined in the diaphragms of E15.5 CAGGS-GCaMP3 in the presence (data not shown) or absence of BHC. Similar to fiber shortening, peak calcium fluorescence increased proportionally with nerve stimulation frequency from 1 to 20 Hz and fatigued in response to 20 Hz (Video 4). In contrast, E15.5 *Vamp2* mutant diaphragms exhibited a dramatically lower level of fiber shortening in response to all frequencies (20 Hz peak length change, *Vamp2* mutant vs. WT; 16.8 ± 8 vs. 160.3 ± 25 μm; *P* < 0.001; *n* = 4 from three mice; Video 3; Figure 7B). In order to determine whether these differences were related to synaptic defects, nerve-evoked muscle APs were recorded in E15.5 *Vamp2* mutants in the presence of BHC. In response to just several pulses of nerve stimulation at 1 Hz, *Vamp2*-deficient muscle fibers exhibited NTF (*n* = 6 from three mice; Figure 7C). This failure was not caused by neurodegeneration, as motor neuron number and motor innervation of diaphragm is exuberant at these stages (data not shown). These studies show that the absence of *Vamp2* severely impairs peripheral neurotransmission during embryogenesis, and likely contributes to the lethality that occurs shortly after birth. Taken together, these data highlight some of the ways by which the skeletal muscle myosin-specific blocker BHC can be applied to study peripheral neurotransmission in both adult and embryonic mice.

## Discussion

These studies illustrate the utility of the skeletal muscle-specific myosin ATPase inhibitor BHC, which paralyzes muscle by impairing the function of the contractile apparatus without affecting neurotransmission. In contrast, previously identified skeletal muscle-specific myosin ATPase blockers such as BTS impair neurotransmission. First, BHC permits the electrophysiological characterization of muscle APs in *ex vivo* intact muscle preparations and in response to nerve as well as muscle stimulation. Second, BHC permits the measurement of NTF in response to high frequencies of nerve or muscle stimulation. We used this pharmacological tool to compare: (i) the rundown of neurotransmitter release (reduced EPP amplitudes), (ii) the extent of muscle fatigue, (iii) changes in calcium dynamics in populations of muscle cells to the onset and extent of NTF in mature and developing NMJs. We utilized this property to show that Vamp2 mutant mice exhibit severe defects in peripheral neurotransmission. Together, we expect that BHC will be a profoundly useful tool for the study of neuromuscular function during development, after injury, and in the context of disease.

Individual nerve-evoked muscle APs recorded in the presence of BHC from the mouse diaphragm exhibit similar morphological attributes to previously published studies of rat diaphragm muscle APs examined in individual muscle fibers in response to direct muscle or nerve stimulation (Delbono and Kotsias, 1987; van Lunteren and Moyer, 1998). Additionally, the recording of muscle APs from transgenic mice expressing GCaMP3 showed that they always correlated with the release of calcium from intracellular stores (data not shown). These results suggested that muscle APs could be measured in intact muscle for the first time in response to HFS, which allows for the empirical demonstration of NTF. We chose to perform an ascending series of tonic phrenic nerve stimulation, since (1) the diaphragm is driven by tonic or sustained stimuli during a variety of conditions, as well as by phasic or cyclic stimulation during respiration, and (2) tonic stimulation paradigms trigger fatigue more quickly than phasic ones (Moyer and van Lunteren, 1999). We found that while adult diaphragm fibers never failed to transduce neural into muscle APs in response to 30-s bouts of stimulation at frequencies of 1–20 Hz, they exhibited NTF in response to 40 and 100 Hz stimulation. When we correlated NTF in the presence of BHC to synaptic depression in the presence of μ-CTX, the onset of NTF coincided with the lowest safety factor estimates (~1.7; Wareham et al., 1994; Wood and Slater, 2001). For example, at 40 Hz, the onset of muscle AP failure occurred between 25 and 27 s, and the onset of EPP amplitude reduction below 1.7-fold occurred at 26–27 s. In response to higher rates of HFS (e.g., 100 Hz), more pronounced percentage rates of muscle AP failure occurred in parallel with more severe drops of EPP amplitude, suggesting that higher safety factor estimates represent concomitantly higher levels of impaired neurotransmission.

Interestingly, we found that NTF occurred in intermittent fashion, with failed APs followed by successful ones. This dynamic nature of muscle AP failure was also confirmed in calcium imaging of transgenic mice, in which fatiguing levels of nerve stimulation produced intermittent flashing of calcium transients (see the 100 Hz video in Video 2; end of 10 and 20 Hz videos in Video 4). Despite this intermittent nature, the percent of successfully transmitted muscle APs gradually fell over time in response to 100 Hz. This decline roughly paralleled the synaptic rundown of ACh observed in the presence of μ-CTX, suggesting an underlying role for the reduction of neurotransmitter release in this phenomenon. However, the “every-other” pattern of muscle AP failure observed from seconds 2 to 5 after 100 Hz stimulation appears to involve other mechanisms, since subsequent recordings from the same cells, in the presence of μ-CTX, showed no such pattern of neurotransmitter release. One possibility is the sensitivity of AChRs, which could be explored by comparing mEPP amplitudes before and immediately after the first few seconds of 100 Hz stimulation. Alternatively, the postsynaptic gating kinetics of Na_*v*_1.4 channels might underlie this unique intermittent pattern of muscle AP failure. While the rate of fast inactivation is unlikely to be changed, since the temporal parameters of muscle APs during this period of 50% failure are similar in magnitude to muscle APs produced by other lower-frequency trains in which there is 100% fidelity (data not shown), the recovery from inactivation, or the deactivation, of these channels may be regulated by HFS, since each of these processes occur within ms (George, 2005). We also observed several subpopulations of muscle fibers based on time to failure in response to 100 Hz. The relative frequencies of these subtypes were correlated to those expressing different MHC isoforms and reflecting different fatigue properties. Finally, in addition to the effects of BHC on muscle predominantly composed of fast-twitch fibers, we observed that BHC was also effective in suppressing contractions in skeletal muscle predominantly composed of slow-twitch fibers as well as cardiac muscle, but not smooth muscle (**Methods** in Supplementary Material).

We next utilized the empirically observed time to NTF to determine its contribution to muscle fatigue. In both tension and optically-measured muscle fiber shortening methods, NTF and fatigue were not observed in response to 30 s of 1 Hz nerve stimulation, consistent with early reports showing an absence of fatigue in response to low-frequency stimulation (Aldrich and Appel, 1985). At higher rates (40 and 100 Hz), there was a proportional relationship between the rate of NTF and decaying muscle responses, consistent with a number of reports providing indirect evidence for a role of NTF in mediating fatigue (reviewed in Sieck et al., 2013). We were intrigued, however, by the finding in diaphragm strips that a loss of tension was produced in response to 10 and 20 Hz stimulation, despite the fact these frequencies failed to cause NTF. However, when we repeated these experiments by measuring fiber shortening in whole diaphragm, we failed to reproduce these results, although there was a non-significant trend for 20 Hz nerve stimulation to cause fatigue, as well as a loss of calcium transient intensity, near the end of the 30-s nerve stimulation period. Thus, in the absence of NTF, at stimulation frequencies between 1 and 20 Hz, muscle fatigue was not produced in whole diaphragm. The discrepancy in results between these two measurements of muscle force could arise from differences in force maintenance between whole vs. strips of diaphragm, or because of the methods used (tension vs. shortening). We favor the former, based on the finding that tension in thin muscle strips results exclusively from forces longitudinal or parallel to each muscle fiber, whereas tension in intact diaphragm is mediated by transverse as well as longitudinal forces (Margulies et al., 1994). The presence of such biaxial forces may accordingly result in a reduced rate of fatigue. Additionally, minor manipulations of muscle length dramatically affect muscle tension and may similarly affect fatigue. Such potential variability in resting length between different fiber strips is less likely to occur in whole diaphragm, in which the entire muscle maintains its insertions into the ribcage and central tendon. Finally, although the phrenic nerve is cut proximally in both muscle strips and whole diaphragm, its branches are also cut in strip preparation, whereas all branches maintain terminal innervation in whole diaphragm. Because motor axons innervating an individual strip are likely part of motor units that also innervate regions which are removed, it is possible that such a preparation impairs the firing rate of motor neurons in response to prolonged HFS.

The immobilization of muscle by BHC provided an opportunity to explore calcium dynamics in muscle, which are altered during muscle fatigue. We took advantage of transgenic mice expressing GCaMP3 in muscle. Although the CAGGS promoter in these mice, composed of CMV and chick β-actin DNA elements, was expected to drive expression in all cell types, we noted that activity-mediated fluorescence in the neuromuscular system was largely restricted to skeletal muscle fibers (data not shown). These responses were also observed in mice expressing conditional GCaMP3 under control of the Myf5-Cre driver, and were not observed in mice expressing GCaMP3 under control of Islet1-Cre or Wnt1-Cre drivers (which drive expression in motor neurons and Schwann cells, respectively; data not shown). Together with the use of BHC, GCaMP3 offers several features that facilitate calcium imaging in intact muscle, including the capacity to be expressed by specific cell types. On the other hand, GCaMP3 exhibits several shortcomings when compared to ratiometric chemical calcium indicators or calcium-selective microelectrodes, such as its inability to estimate quantitative calcium concentrations (see however Albantakis and Lohmann, 2009). Additionally, the low equilibrium dissociation constant (K_D_) of GCaMP3 (287 nM) for calcium makes this molecule unsuitable for the measurement of fast calcium events such as the rate of calcium release. Although GCaMP3 can only reliably represent entire transients up to 6 Hz, based on limitations in the speed of rise and decay times within this sensor (Tian et al., 2009), newer variants such as GCaMP6f exhibit resolvable changes in calcium fluorescence at higher frequencies (Chen et al., 2013). For the present study, only the magnitude and maintenance of peak transient amplitudes of were required to determine the effect of fatiguing nerve stimulation. We found that similar to fiber shortening, 1–20 Hz nerve stimulation failed to produce significant changes in calcium transient intensity in muscle, whereas NTF-causing frequencies produced a loss of signal similar to (40 Hz) or more pronounced than (100 Hz; see Figure 6E) the loss of force caused by these stimuli.

Lastly, the use of BHC allows for the first time the measurement of synaptic transmission in embryonic mice, which are insensitive to Na_v_1.4 antagonists (Lupa et al., 1993; Wilson et al., 2011). Although the use of bathing solutions with low calcium allows nerve stimulation to produce subthreshold transients that can be compared between different samples (data not shown), the effect on presynaptic function of low calcium precludes the analysis of presynaptic function on neural transmission. Subparalytic doses of curare can also produce subthreshold endplate potentials that can be recorded electrophysiologically at the embryonic NMJ, but the presence of off-target effects on presynaptic terminals (Glavinović, 1979), coupled with the difficulty in generating reliable effects, make this perturbation unfavorable. In any case, each of these manipulations measures neural transmission at the embryonic NMJ only by the subthreshold EPP, whereas BHC allows for the study of muscle APs and subsequent calcium dynamics in response to single nerve stimuli or HFS. In order to demonstrate the nerve stimulation frequency that was sufficient to induce fatigue, the optical measurement of shortening was applied, as conventional tension transducing equipment is excessively large for embryonic diaphragm fibers. Using this optical technique, we were able to establish that 30 s of 20 Hz nerve stimulation produced a profound fatigue or inability to maintain maximal muscle fiber shortening in E15.5 diaphragm. Calcium imaging of diaphragms from CAGGS-GCaMP3 mice replicated these findings and also provided evidence that fatiguing muscle fibers rebound before ultimately failing completely. Finally, we provide evidence that nerve stimulation in *Vamp2* mutants can produce individual muscle APs, but leads to NTF almost immediately at frequencies as low as 1 Hz. Taken together, these studies suggest that BHC represents a powerful new tool for measuring multiple aspects of neuromuscular function in adult and embryonic mice, such as the correlation between neurotransmitter release and neurotransmission success rate, the correlation between neurotransmission success rate and muscle tension, and the correlation between muscle calcium handling and muscle tension, in a wide variety of mouse models of neuromuscular dysfunction and/or disease.

## Author contributions

DH designed, carried out, and interpreted the experiments in Figures 1–7. DS carried out, interpreted and drafted the writing of the tension experiments in Figure 4. SM carried out, interpreted, and drafted the writing of the immunohistochemical experiments in Figure 3. GWH analyzed, interpreted, drafted, and revised the writing of the experiments in Figures 5–7. TWG designed and interpreted the experiments in Figures 1–7. DH drafted and TWG edited the manuscript. All authors approve of the final version and agree to be held accountable for all aspects of the work.

### Conflict of interest statement

The authors declare that the research was conducted in the absence of any commercial or financial relationships that could be construed as a potential conflict of interest.

## Supplementary material

Supplementary Figure 1 **BTS blocks muscle movement but also disrupts neurotransmission in the adult mouse diaphragm**. Fifty μM of BTS was added to hemidiaphragm preparations and muscle potentials were recorded in response to 1 Hz phrenic nerve stimulation with sharp intracellular electrodes. Recorded muscle potentials failed to exceed several mV.

Video 1 **Nerve stimulation at frequencies that fail to cause NTF in the adult fail to cause fatigue**. Thirty-second brightfield videos of tonic phrenic nerve stimulation of adult diaphragm at 1, 10, 20, 40, and 100 Hz. Note development of tetanic contraction over time in response to 10 Hz.

Video 2 **Nerve stimulation at frequencies that fail to cause NTF in the adult fail to cause a loss of calcium transient intensity**. Thirty-second fluorescence videos of tonic phrenic nerve stimulation of adult diaphragm at 1, 10, 20 40, and 100 Hz. Note flashing of individual fibers during first half of 100 Hz stimulation, representing intermittent neurotransmission.

Video 3 **Development of fatigue in response to high-frequency nerve stimulation (HFS) in embryonic WT and *Vamp2* mutant diaphragm**. Thirty-second fluorescence videos of tonic phrenic nerve stimulation of the left side of E15.5 diaphragm at 1, 5, 10, and 20 Hz in WT (top panels) and *Vamp2* mutant (bottom panels) mice. Note the failure in WT diaphragm to maintain peak shortening in response to 20 Hz stimulation. Note also the failure of *Vamp2* mutant diaphragm to maintain the response to even 1 hz stimulation.

Video 4 **Development of fatigue in response to HFS in embryonic WT diaphragm expressing GCaMP3**. E15.5 CAGGS-GCaMP3 mice were stimulated in the absence of BHC to illustrate the concordance of fiber shortening and calcium transient intensity. Note the progressive loss in maintenance of peak calcium transient intensity in response to increasing nerve stimulation frequency. Note also that despite the ability of diaphragm to maintain peak or near-peak shortening in response to 10 Hz for the entire 30-s period, an undulating pattern of intermittent fluorescence occurs toward the end of stimulation, which appears to presage fatigue, because this pattern of fluorescence occurs earlier and more profoundly in response to 20 Hz stimulation. Finally, note the “spontaneous” activation of bands of muscle fibers in all conditions before nerve stimulation, likely representing the spontaneous release of transmitter that occurs in response for several hours after nerve severance.

### Methods: effects of BHC on slow-twitch skeletal and cardiac muscle

Based on the heterogeneous nature of fiber subtype in the diaphragm, together with the observation that movement is suppressed within all muscle cells of the diaphragm in response to BHC, that seems to be no predilection of this drug for fast vs. slow muscle fibers. Moreover, muscle cells with different failure rates in response to 100 Hz stimulation were recorded from, suggesting that muscle cells expressing different MHC isoforms are all sensitive to BHC. To further examine this feature, the soleus was dissected and the nerve stimulated by suction electrode before and after 30 min of 50 μM BHC treatment and 30 min of perfused Krebs-Ringer's solution (washout). By visual observation, contractions in the majority of the soleus were blocked, but a small region on the lateral half still exhibited movement. After a further incubation with 100 μM BHC for 30 min and washout for 30 min, all movement of the soleus desisted. Control soleus muscles still exhibited twitch in response to nerve stimulation after a comparable time. Current studies are investigating whether the region that remained after the first dose were enriched in slow fibers, as the mouse soleus is composed only of ~40% slow-twitch fibers (Wigston and English, 1992).

In order to examine the effects on the heart, the left ventricle was dissected and the papillary muscles were exposed. In response to electrical field stimulation (EFS), contractions could be obtained for several hours. After 30 min of 50 μM BHC treatment and washout, this EFS-induced twitch disappeared, whereas in control papillary muscle, it persisted. These results suggest that BHC, in contrast to BTS, suppresses contraction in cardiac muscle and slow-twitch skeletal muscle, as well as fast-twitch skeletal muscle. However, when strips of circular smooth muscle-containing large intestine were induced to contract via mucosal stimulation, 50–100 μM BHC failed to suppress these movements.

## Abbreviations

NMJ: Neuromuscular Junction
MN: Motor neuron
NTF: Neural Transmission Failure
RRP: Ready Releasable Pool
AP: Action Potential
EPP: Endplate Potential
mEPP: miniature Endplate Potential
Ach: Acetylcholine
μ-CTX: μ-conotoxin
BHC: 3-(N-butylethanimidoyl)-4-hydroxy-2H-chromen-2-one
BTS: N-benzyl-p-toluene sulphonamide
HFS: High-frequency Nerve Stimulation
MHC: Myosin Heavy Chain
ST: Spatio-temporal.
