# Supplementary figures and images for: A Novel Striated Muscle-Specific Myosin-Blocking Drug for the Study of Neuromuscular Physiology
Source: Front Cell Neurosci. 2016 Dec 1;10:276. doi: 10.3389/fncel.2016.00276 (PMC5130989; doi:10.3389/fncel.2016.00276)

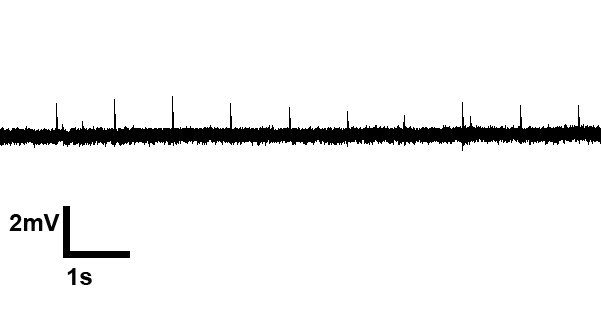

Supplement: Supplementary Figure 1 — BTS blocks muscle movement but also disrupts neurotransmission in the adult mouse diaphragm. Fifty μM of BTS was added to hemidiaphragm preparations and muscle potentials were recorded in response to 1 Hz phrenic nerve stimulation with sharp intracellular electrodes. Recorded muscle potentials failed to exceed several mV. [file Image1.JPEG]
